# Laparoscopic repair combined with hysteroscopy of cesarean section scar after cesarean scar pregnancy resulting in a live birth: A case report

**DOI:** 10.20407/fmj.2024-003

**Published:** 2024-10-31

**Authors:** Kyohei Takada, Eiji Nishio, Kiriko Kotani, Arata Kobayashi, Akiko Owaki, Yoshiteru Noda, Mayuko Ito, Hironori Miyamura, Haruki Nishizawa

**Affiliations:** Department of Obstetrics and Gynecology, Fujita Health University, School of Medicine, Toyoake, Aichi, Japan

**Keywords:** Cesarean scar syndrome, Cesarean scar disorder, Laparoscopic surgery, Post cesarean scar defect, Cesarean scar pregnancy

## Abstract

We report a case where a spontaneous pregnancy and delivery of a live baby were achieved after laparoscopic repair combined with hysteroscopy of the cesarean section scar secondary to treatment for a prior cesarian section scar pregnancy. [Case] 31 years old, three pregnancies, and one delivery. A spontaneous abortion. Her first child was delivered by elective cesarean section due to pelvic position (breech presentation). During her second pregnancy, she was diagnosed with cesarean section scar pregnancy and underwent dilation and curettage. Subsequently, a laparoscopic repair of the uterine scar was performed using hysteroscopy owing to thinning of the cesarean section scar area. At six months postoperative, she conceived spontaneously and delivered a live baby by elective cesarean section at 38 weeks 2 days gestation. Conclusion: Thus, laparoscopic repair combined with hysteroscopy of the uterine scar can be a useful treatment option for patients with uterine scarring secondary to cesarean section scar pregnancy.

## Introduction

Cesarean scar syndrome (CSS) is a disease concept proposed by Morris et al. in 1995 in which a depressed, thinning scar forms at the site of hysterotomy after a C-section, causing abnormal menstruation and secondary infertility due to blood retention at the site.^1^ More recently, Saskia et al.^2^ associated a series of symptoms caused by cesarean section scarring and proposed the term “cesarean scar disorder” (CSDi). Low-dose estrogen/progesterone combinations^3^ may be useful as a conservative treatment for hemorrhage due to these illnesses, but hormonal treatment is not a radical solution for patients who wish to have children, in which case uterine scar repair may be the surgical treatment of choice.

Conversely, cesarean scar pregnancy (CSP) is an ectopic pregnancy that implants in the cesarean section scar, and it is a rare disease that occurs in 0.45% of all pregnancies after previous cesarean section.^4^

We report a rare case in which a patient with CSDi secondary to CSP underwent laparoscopic repair combined with hysteroscopy of the uterine scar, followed by spontaneous pregnancy.

## Case

### Current medical history:

**Figure 1:**
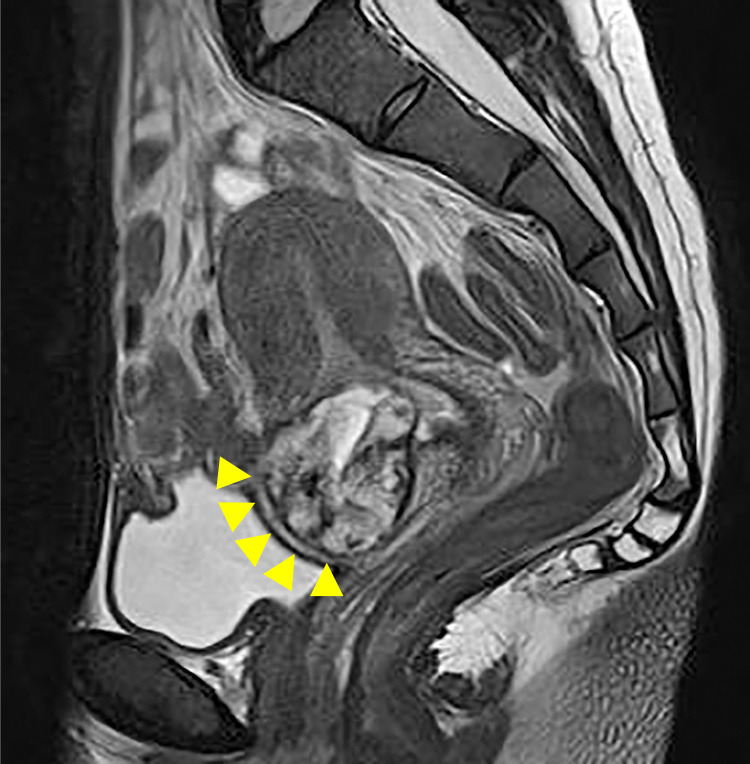
Simple MRI T2-weighted sagittal section: cesarean scar pregnancy (CSP) findings (arrowhead).

**Figure 2:**
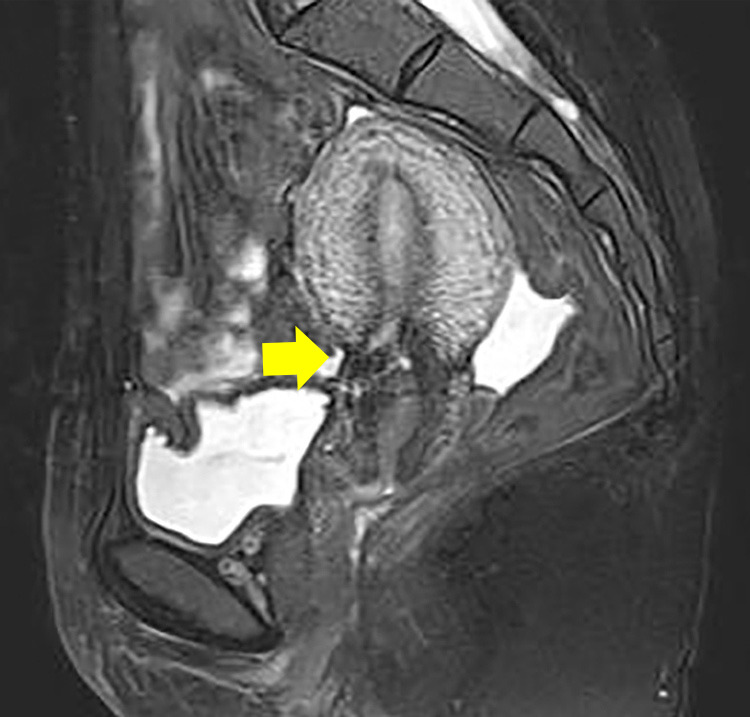
Sagittal simple MRI T2-weighted image: cesarean scar disorder; residual myometrial thickness (RMT) area in CSDi (arrows).

31 years old. Three pregnancies and one miscarriage (Spontaneous abortion). Five years ago, she delivered her first child through elective cesarean section due to pelvic position (breech presentation). A year later, a fetal sac was observed at the site of a previous incision and she was diagnosed with CSP (Figure 1). Methotrexate (MTX) therapy was performed, but the gestational sac remained, thus dilation and curettage was performed using hysteroscopy. Ultrasound depicting thinning and wedging of the cesarean section scar site after dilation and curettage along with chronic pelvic pain was often observed during follow-up. Residual myometrial thickness (RMT) measured by magnetic resonance imaging performed 2 years after CSP was 6 mm (Figure 2). She did not become pregnant for 3 years after the onset of CSP, and although RMT was maintained, chronic pelvic pain and secondary infertility fulfilled the criteria for CSDi. Considering the possibility of recurrent CSP, a laparoscopic repair combined with hysteroscopy of the uterine scar was performed.

### Surgical findings:

**Figure 3:**
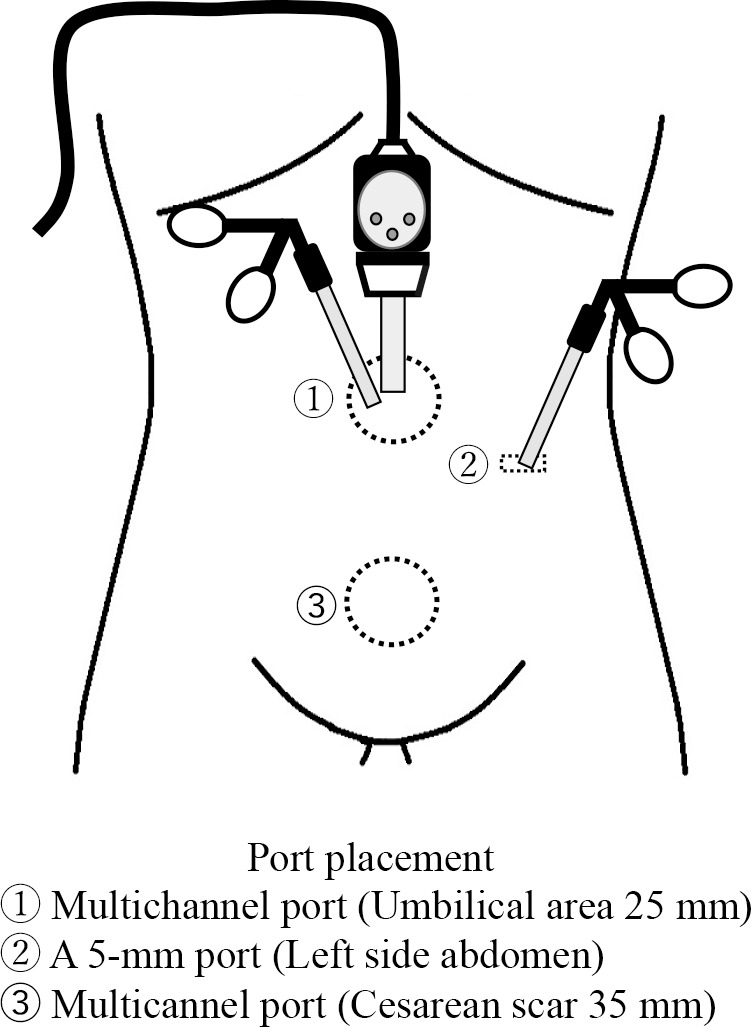
Surgical port placement.

**Figure 4:**
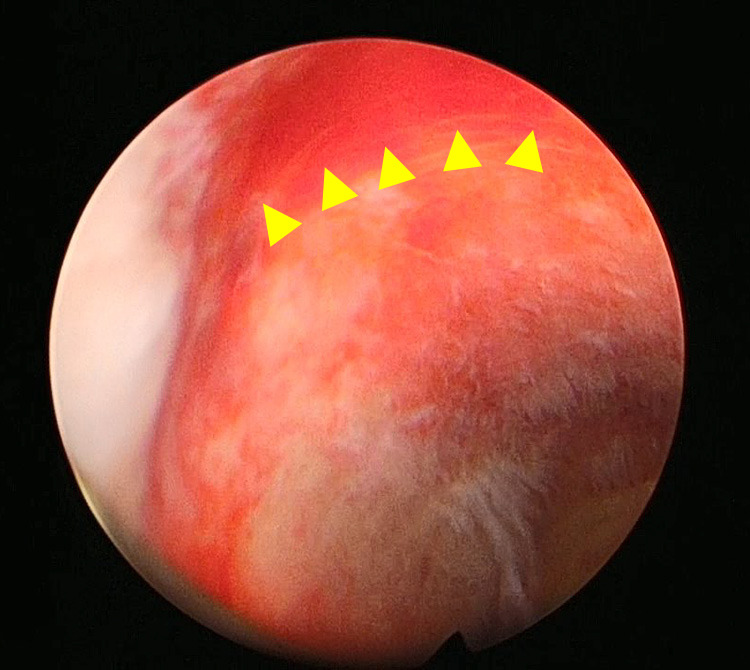
Hysteroscopic findings: Areas of thinning (arrows)

**Figure 5:**
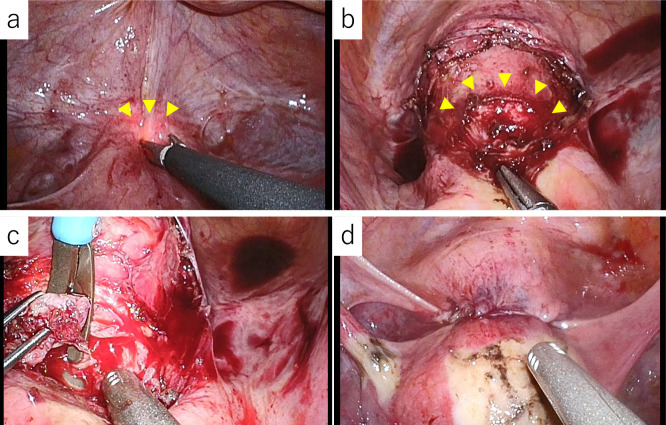
Laparoscopic findings. a. Identification of thinning areas using the light source of the hysteroscope (arrowhead). b. Scarred area after serosal detachment (arrowhead). c. Myotomy from above pubis. d. Findings after uterine scar repair.

Surgery time was 3 hours and 31 minutes, and blood loss was 77 g. A multichannel port in the umbilicus and a 5 mm port were placed in the left side of the abdomen. Additionally, a 35 mm skin incision was made over the cesarean section incision scar above the pubic bone, and a multichannel port was placed (Figure 3). A 5-mm flexible scope was used. There was no evidence of adhesion in the abdominal cavity, but the cesarean section scar was covered by the peritoneum of vesicouterine pouch, and the thinned area could not be identified. Therefore, we inserted a hysteroscope and directly observed the uterine lumen and identified a thinned area, although there was no scar depression or obvious atypical vessels (Figure 4). The thinning of the uterus was confirmed laparoscopically through the light source of the hysteroscope (Figure 5-a), and the extent of resection was determined by palpating the uterus directly through the suprapubic opening and palpating the depression at the same site. The peritoneum of vesicouterine pouch was dissected to expose the depressed thinning site (Figure 5-b), and a scissors and forceps was inserted from above the pubis to debride the thinning site into a wedge shape (Figure 5-c), followed by suturing of two layers of myometrium. The first layer was sutured with No. 2-0 synthetic resorbable thread, and the second layer was sutured with No. 0 synthetic resorbable thread. The detached peritoneum was sutured closed (Figure 5-d). After cauterizing the endometriosis lesion in the pelvis, the operation was concluded by application of an anti-adhesion agent.

### Postoperative course:

**Figure 6:**
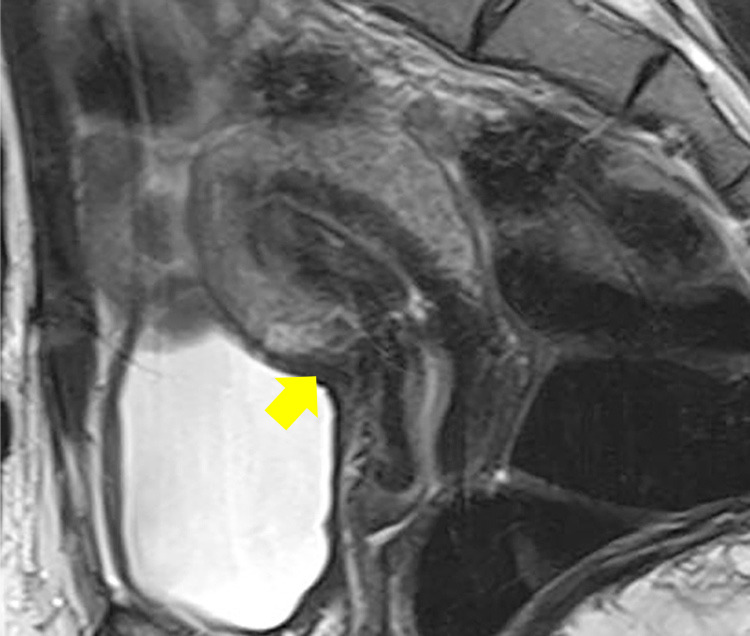
Simple MRI T2-weighted sagittal section: Residual myometrial thickness (RMT) 4 months after uterine scar repair (arrow)

The patient progressed well and was discharged on the fourth postoperative day. The RMT of the repair site on MRI performed 6 months after repair surgery was 13 mm (Figure 6).

**Figure 7:**
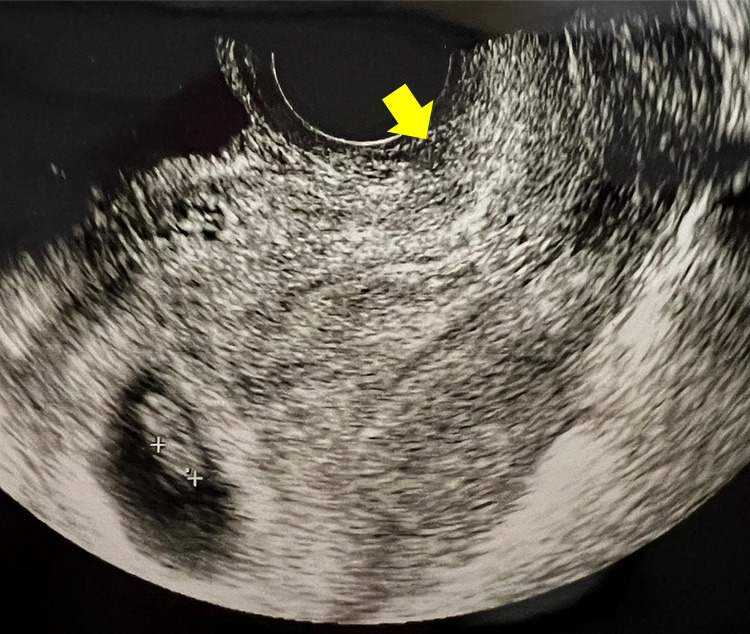
Ultrasound: RMT area during spontaneous pregnancy (arrow)

At 8 months post-surgery, the patient became pregnant spontaneously and there was no evidence of re-thinning of the repaired scar when a transvaginal ultrasonography showed a fetal sac in the uterine body (Figure 7). There were no symptoms of threatened uterine rupture during the pregnancy, and the fetus was developing well.

The patient underwent elective cesarean section at 38 weeks and 2 days of pregnancy, and at the time of laparotomy no thinning of the repaired scar area was observed; moreover, a transverse incision of the lower uterine segment was possible. The birth weight of the newborn was 2945 g, Apgar score 8/9 points (1 min/5 min), umbilical artery blood gases 7. 246, BE-5.3. Postoperative course was good and no CSDi symptoms were observed after cesarean section.

## Discussion

In recent years, the number of cesarean sections in Japan has been increasing, and the number of CSDi cases is expected to increase as well. Accordingly, the demand for fertility-sparing surgery for patients who wish to have children is expected to increase. There are reports of increased pregnancy rates after cesarean section scar repair as a fertility-sparing procedure for CSDi.^5^ There have been reports of abdominal and hysteroscopic cesarean section scar repair,^6^ and recently laparoscopic uterine scar repair has also been performed.^7^ Conversely, CSP is a rare ectopic pregnancy that implants in the cesarean section scar.^4^ There have been reports of CSDi cases secondary to CSP but they are extremely few in number, and no treatment has been established.

Although clear criteria for cesarean section scar repair for CSDi is available, Tanimura et al.^7^ indicated surgery if the RMT was 2.5 mm or less in addition to the presence of infertility, and Tsuji et al.^8^ indicated hysteroscopic surgery for RMT of 2.2 mm or less; as such, the RMT of 6 mm in this case did not meet the surgical criteria reported to date. However, Morlando et al.^9^ reported that 17.6% of CSP patients had recurrent CSP, although there is no clear evidence that uterine scar repair prevents CSP, we decided to perform uterine scar repair in the hope that it would be effective.

**Figure 8:**
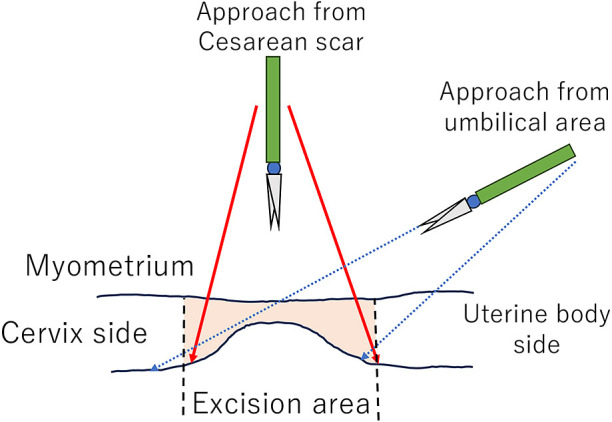
Different approach angles (schema) for different port placements for scar a resection maneuvers.

The operation was performed laparoscopically with a hysteroscope in case the cesarean section scar site could not be identified. Although it was not possible to visually determine the depressed area laparoscopically or hysteroscopically, it was possible to identify the thinning area by light transparency, and direct contact with the area was useful in determining the extent of resection of the thinning area. We believe that the suprapubic approach for excision of the scar allowed for more accurate identification and excision of the scar compared to the umbilical approach (Figure 8). Scissors forceps was used for excision of the scar to avoid the use of energy devices as much as possible and to prevent suture insertion failure due to thermal injury of the muscle tissue. Suture insertion of the muscle layer was performed manually through a multichannel port on the pubic bone. Suture insertion of a cesarean section scar requires high skill in needle placement with the common diamond placement due to anatomical reasons. However, use of a multichannel port on the pubic bone as shown in the present study enables precise suture insertion to the muscle layer by manual needle manipulation and ligation.

In this case, the suture insertion thread was a two-layer suture with a single ligation using synthetic resorbable braided thread. However, the discussion on suture insertion has been reported in various cases of cesarean section. For example, there have been comparisons between single and continuous suture insertion, and between one-layer and two-layer suture insertion, but there is generally no difference in the long-term prognosis.^10^ However, there are reports that one-layer suture insertion decreases RMT compared to two-layer suture insertion, and that this tendency is more marked for continuous suture insertion,^11^ that suture insertion increases the risk of placenta accreta adhesion compared to single suture insertion,^12^ and that one-layer suture insertion increases the risk of uterine rupture compared to two-layer sutures (suture insertion).^13^ Therefore, in this case, a two-layer suture with a single ligation suture insertion was performed to ensure blood flow in the muscle layer and to prevent suture insertion failure and re-thinning due to tension reduction. On the other hand, novel suture insertion threads have been introduced in recent years, and the usefulness of continuous suture insertion using suture insertion threads is an issue for future study.

In this case, there was no postoperative suture insertion failure or muscle layer re-thinning, and the patient had a good course after a normal pregnancy. Although more cases are needed to prove the usefulness of laparoscopic repair combined with hysteroscopy of the uterine scar in the lower uterine segment, it is expected to be a potential option for fertility-sparing surgery.

## Conclusions

A laparoscopic repair combined with hysteroscopy for CSDi secondary to CSP may be a viable option. Additionally, the use of a hysteroscope and the addition of a small laparotomy near the scar site can improve the precision of the surgical operation.

Conflict of Interest: The authors have no conflict of interest to disclose in relation to this paper.

## Supplementary Material

PDF-Japanese
