# Supplementary material for: Laparoscopic repair combined with hysteroscopy of cesarean section scar after cesarean scar pregnancy resulting in a live birth: A case report
Source: Fujita Med J. 2024 Oct 31;11(1):48–51. doi: 10.20407/fmj.2024-003 (PMC11782943; doi:10.20407/fmj.2024-003)

症例報告

帝王切開瘢痕部妊娠後に続発した帝王切開子宮瘢痕症に対して子宮鏡併用腹腔鏡下子宮瘢痕部修復術により生児を得た１例

高田　恭平(M.D.)　西尾　永司(M.D. Ph.D.)　小谷　燦璃古(M.D.)　小林　新(M.D.)　大脇　晶子(M.D. Ph.D.)　野田　佳照(M.D. Ph.D.)　伊藤　真友子(M.D. Ph.D.)　宮村　浩徳(M.D. Ph.D.)　西澤　春紀(M.D. Ph.D.)
藤田医科大学　医学部　産婦人科学

ランニングタイトル: A Case of Successful Delivery After Repair for Cesarean Scar Disorder

責任著者氏名：高田　恭平
住所：愛知県豊明市田楽が窪 1-98　藤田医科大学病院産婦人科
電話番号：0562-93-9294
メールアドレス：kyou-t@fujita-hu.ac.jp

抄録

【緒言】帝王切開瘢痕部妊娠の治療後に続発した帝王切開子宮瘢痕症に対し、子宮鏡併用腹腔鏡下子宮瘢痕部修復術を施行し、自然妊娠で生児を得た症例を経験したので報告する。【症例】31 歳、3 妊 1 産。自然流産 1 回。第 1 子を骨盤位のため選択的帝王切開で出産した。2 回目の妊娠時に帝王切開瘢痕部妊娠と診断し、子宮内容除去術を行った。その後、帝王切開瘢痕部の菲薄化を認めたため、子宮鏡併用腹腔鏡下子宮瘢痕部修復術を施行した。術後 6 か月に自然妊娠し、妊娠 38 週 2 日に選択的帝王切開術で生児を得た。【結語】帝王切開瘢痕部妊娠後に続発した帝王切開子宮瘢痕症に対する子宮鏡併用腹腔鏡下子宮瘢痕部修復術は有用な治療の選択肢となり得る。

キーワード：帝王切開瘢痕症候群、帝王切開子宮瘢痕症、腹腔鏡下子宮瘢痕部修復術、帝王切開瘢痕部妊娠、子宮鏡

【緒言】

帝王切開瘢痕症候群(cesarean scar syndrome; CSS)は 1995 年に Morris らにより提唱された疾患概念で、帝王切開後の子宮切開部に陥凹性の菲薄化した瘢痕を形成し、同部位に血液が貯留し月経異常や続発性不妊をきたす疾患である[1]。さらに近年、Saskia ら[2]が帝王切開瘢痕に起因する一連の症状を関連付け、帝王切開子宮瘢痕症(cesarean scar disorder; CSDi)を提言している。これらの疾患群による不正出血に対する保存的治療として低用量エストロゲン・プロゲステロン配合薬[3]が有用であるが、ホルモン治療は挙児希望を有する患者に対する根本的な解決にはならず、外科的治療として子宮瘢痕部修復術が選択されうる。

一方、帝王切開瘢痕部妊娠(cesarean scar pregnancy ; CSP)は帝王切開瘢痕部に着床する異所性妊娠であり、既往帝王切開後妊娠の 0.45%に発生する稀な疾患である[4]。

今回、CSP の治療後に続発した CSDi 患者に対して、子宮鏡併用腹腔鏡下子宮瘢痕部修復術を施行した後、自然妊娠にて生児を得た貴重な症例を経験したので報告する。

【症例】

現病歴：

31 歳。3 妊 1 産（自然流産 1 回）。5 年前に第 1 子を骨盤位のため選択的帝王切開で出産した。4 年前、前回切開創と思われる部位に胎嚢を認め CSP と診断し(figure1)、Methotrexate(MTX)療法を施行したが、胎嚢が残存したため子宮鏡による子宮内容除去術を実施した。子宮内容除去術後から帝王切開瘢痕部の菲薄化と楔状変化を超音波で認め、経過観察中に慢性的な骨盤痛がしばしば確認された。CSP から 2 年後に施行した MRI で計測した残存子宮筋層厚(residual myometrial thickness ; RMT)は 6 ㎜であった(figure2)。CSP 発症から 3 年経過しても妊娠しないため、RMT は保たれているものの慢性的な骨盤痛と二次性の不妊症から CSDi の基準を満たすことや、CSP を繰り返す可能性があることを考慮し、子宮鏡併用腹腔鏡下子宮瘢痕部修復術を施行した。

手術所見：

手術時間 3 時間 31 分、出血量 77g、ポート配置は臍にマルチチャネルポートを装着し、左側腹部に 5 ㎜ポートを配置した。加えて恥骨上の帝王切開皮膚切開痕にも 35 ㎜の皮膚切開を加え、マルチチャネルポートを装着した(figure3)。スコープは 5 ㎜の軟性鏡を用いた。腹腔内に癒着所見はなかったが、帝王切開瘢痕部は膀胱子宮窩腹膜で覆われ、菲薄部位の同定はできなかった。そこで、子宮鏡を挿入し子宮内腔を直接観察すると、瘢痕部の陥凹や明らかな異型血管はなかったが、菲薄部位を同定した(figure4)。子宮の菲薄部を子宮鏡の光源を透見して腹腔鏡下で確認するとともに（figure5-a)、恥骨上の開創部から直接子宮を触診して同部位の陥凹を触知することで切除範囲を決定した。膀胱子宮窩腹膜を剥離して陥凹した菲薄部位を露出し（figure5-b)、恥骨上からハサミ鉗子を挿入して菲薄部位を楔状にデブリードメントした後に(figure5-c)、子宮筋層を 2 層縫合した。1 層目は 2-0 号合成吸収糸にて単結紮縫合し、2 層目は 0 号合成吸収性編糸を用いて同様に単結紮縫合した。剥離した腹

膜は連続縫合で閉鎖した(figure5-d)。骨盤内の子宮内膜症病変を焼灼後、癒着防止剤を貼付して手術を終了とした。

術後経過：

経過は良好で術後 4 日目に退院となった。修復術後 6 か月に施行した MRI の修復部位の RMT は 13 ㎜であった(figure6)。

術後 8 か月後に自然妊娠に至り、経腟超音波検査で子宮体部に胎嚢を確認した際にも、瘢痕部修復部の再菲薄化は認めなかった(figure7)。妊娠経過中に切迫子宮破裂の症状はなく、胎児の発育も順調であった。

妊娠 38 週 2 日に選択的帝王切開とし、開腹時の肉眼所見で瘢痕修復部の菲薄化はなく、子宮下節横切開が可能であった。出生児は 2945g、Apgar score : 8/9 点(1 分/5 分)、臍帯動脈血液ガス 7.246、BE-5.3 であった。術後経過は良好で、帝王切開後も CSDi の症状は認めていない。

【考察】

近年、日本における帝王切開術件数は増加しており、CSDi の症例数も増加すると考えられる。それに伴い、挙児希望患者に対する妊孕性温存手術の需要は高くなると予想される。CSDi に対する妊孕性温存手術として帝王切開瘢痕部修復術が行われ、妊娠率が増加したとする報告がある [5)]。これまで腹式帝王切開瘢痕部修復術や子宮鏡下術の報告 [6)] があるが、近年では腹腔鏡下子宮瘢痕部修復術も行われるようになってきている [7)]。一方、帝王切開瘢痕部妊娠(cesarean scar pregnancy ; CSP)は帝王切開瘢痕部に着床する稀な異所性妊娠であり [4)]、CSP 後に続発した CSDi 症例の報告は少なく、その治療法は確立されていない。

CSDi に対する帝王切開瘢痕部修復術の適応について明確な基準はないが、Tanimura ら [7)] は、不妊症状に加え、RMT が 2.5 ㎜以下の場合は手術適応とし、また Tsuji ら [8)] は RMT が 2.2mm 以下で子宮鏡手術の適応としているが、本症例の RMT は 6 ㎜でありこれまでの報告における手術基準は満たしていない。しかし、Morlando ら [9)] は 17.6%の CSP 患者が CSP を繰り返したと報告しており、子宮瘢痕部修復術が CSP を予防するという明確なエビデンスはないものの、修復術による CSP の再発予防効果に期待して子宮瘢痕部修復術を決定した。

手術は内視鏡視下から帝王切開瘢痕部が確認できない場合に備えて子宮鏡を併用した。内視鏡下でも子宮鏡下でも陥凹部分を視覚的に判断することはできなかったが、光の透見によって菲薄化部位の同定が可能であり、同部位を直接触知することで菲薄化部位の切除範囲の決定に有用であった。瘢痕部の切除は恥骨上からアプローチすることで、臍からのアプローチと比較して瘢痕部に対し、より正確な同定と切除が可能であったと考えている(figure.8)。また、瘢痕部の切除にはハサミ鉗子を用いることでエネルギーデバイスの使用を極力回避し、筋組織の熱損傷による縫合不全を予防することを目的とした。筋層の縫合は

恥骨上のマルチチャネルポートからアプローチし用手的に行った。帝王切開瘢痕部の縫合は、解剖学的な理由から一般的なダイヤモンド配置では、運針に高い技術が要求されるが、今回のように恥骨上にマルチチャネルポートを装着することで用手的な運針と結紮による精細な筋層縫合を可能とした。

本症例では、縫合糸は合成吸収性編糸を用いて単結紮の 2 層縫合としたが、子宮筋層の縫合に関する議論は、帝王切開において様々な報告がされている。例えば単縫合と連続縫合の比較や 1 層縫合と 2 層縫合の比較がされているが概ね長期予後に差はないとされる [10)]。しかし、1 層縫合が 2 層縫合と比較し RMT を減少させ、連続縫合でその傾向が強くなるとする報告や [11)]、連続縫合は単縫合と比較し癒着胎盤リスクを増加させるとする報告 [12)]、また 1 層縫合は 2 層縫合と比較し子宮破裂リスクが高いとする報告も存在する [13)]。そのため本症例は単結紮の 2 層縫合を行い、筋層の血流の確保と減張による縫合不全や再菲薄化防止を図った。一方、近年、新たに有棘縫合糸が登場しており、有棘縫合糸を使用した連続縫合の有用性について、今後の検討課題と考えている。

本症例では術後の縫合不全や筋層の再菲薄化は認めず、正常妊娠後の経過も良好であった。今回の子宮鏡下併用腹腔鏡下子宮瘢痕部修復術の有用性の証明は今後も症例の蓄積が必要であるが、妊孕性温存性手術の選択肢の一つとして期待できる。

【結語】

CSP 後に続発した CSDi に対する子宮鏡併用腹腔鏡下子宮瘢痕部修復術は有効な選択肢となり得る。加えて子宮鏡を併用することや瘢痕部近くに小開腹を加えることは手術操作の精度向上に寄与する。

【利益相反】この論文に関連して開示すべき利益相反状態にはありません。

【謝辞】本論文を執筆するにあたり指導してくださった医局員の方々に感謝の意を表する。

参考文献

1） Morris H. Surgical pathology of the lower uterine segment caesarean section scar: is the scar a source of clinical symptoms? Int J Gynecol Pathol 1995 ; 14:16-20.
2） Klein Meulemen S, Murji A, van den Bosch T, Donnez O, Grimbizis G, Saridogan E, Chantraine F, Bourne T, Timmerman D, Huirne J, de Leeuw R. Definition and Criteria for Diagnosing Cesarean Scar Disorder. JAMA Netw Open 2023;6
3） Tahara M, Shimizu T, Shimoura H. Preliminary report of treatment with oral contraceptive pills for intermenstrual vaginal bleeding secondary to a cesarean section scar. Fertil Steril 2006; 86:477-9.
4） Petersen KB, Hoffmann E, Larsen CR, Nielsen HS． Cesarean scar pregnancy: a systematic review of treatment studies. Fertil Steril 2016; 105:958-67.
5） Tsuji S, Murakami T, Kimura F, Tanimura S, Kudo M, Shozu M, Narahara H, Sugino N. Management of secondary infertility following cesarean section: Report from the Subcommittee of the Reproductive Endocrinology Committee of the Japan Society of Obstetrics and Gynecology. J Obstet Gynaecol Res 2015; 41: 1305-12.
6） Vrijdagahs V, Dewilde K, Froyman W, Van den Bocsh T. Hysteroscopic management of caesarean scar defects. J Obstet Gynaecol 2022; 42:816-22.
7） Tanimura S, Funamoto H, Hosono T, Shitano Y, Nakashima M, Ametani Y, Nakano T. New diagnostic criteria and operative strategy for cesarean scar syndrome: Endoscopic repair for secondary infertility caused by cesarean scar defect. J Obstet Gynaecol Res 2015; 41:1363-9.
8） Tsuji S, Nobuta Y, Yoneoka Y, Nakamura A, Amano T, Takebayashi A, Hanada T, Murakami T. Indication Criteria of Hysteroscopic Surgery for Secondary Infertility due to Symptomatic Cesarean Scar Defect Based on Clinical Outcomes: A Retrospective Cohort Study. J Minim Invasive Gynecol 2023;30:576-81.
9） Morlando M, Buca Danilo, Timor-Tritsch I, Cali G, Palacios-Jaraquemada J, Monteagudo A, Khalil A, Cennamo C, Manna VL, Liberati M, D’Amico A, Nappi L, Colacurci N, D’Antonio F. Reproductive outcome after cesarean scar pregnancy: A systematic review and meta-analysis. Acta Obstet Gynecol Scand 2020; 99: 1278-89.
10) Abalos E, Addo V, Brocklehurst P, EI Sheikh M,Farrell B, Gray S, Hardy P, Juszczak E, Mathews JE, Naz Masood S, Oyarzun E, Oyieke J, Sharma JB, Spark P. Caesarean section surgical techniques (CORONIS): a fractional, factorial, unmasked, randomised controlled trial. Lancet 2013; 382: 234-48.
11) Roberge S, Demers S, Girard M, Vikhareva O, Maekey S, Chaillet N, Moore L, Paris G, Bujold E. Impact of uterine closure on residual myometrial thickness after cesarean: a randomized controlled trial. Am J Obstet Gynecol 2016; 214: 507.e1-507.e6.

12) Sumigawa S, Sugiyama C, Kotani T, et al. Uterine sutures at prior caesarean section and placenta accreta in subsequent pregnancy: a case-control study. BJOG 2014; 121:866-74; discussion 875.
13) Bujold E, Bujold C, Hamilton EF, Harel F, Gauthier RJ. The impact of a single-layer or double-layer closure on uterine rupture. Am J Obstet Gynecol 2002; 186: 1326-30.

Figure.1　単純 MRI T2 強調像矢状断：cesarean scar pregnancy (CSP)所見（矢頭）

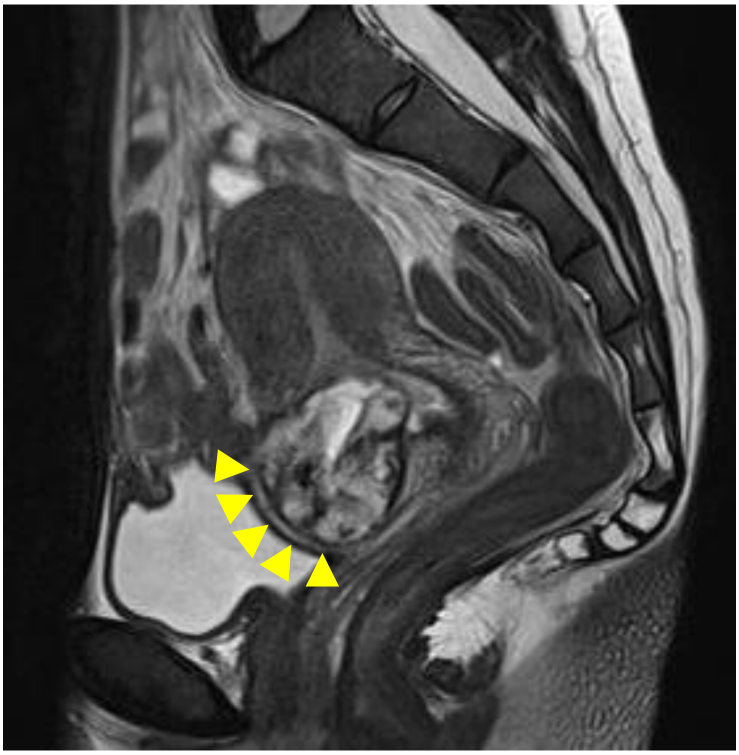

Fifure.2　単純 MRI T2 強調像矢状：cesarean scar disorder; CSDi における residual myometrial thickness (RMT)部（矢印）

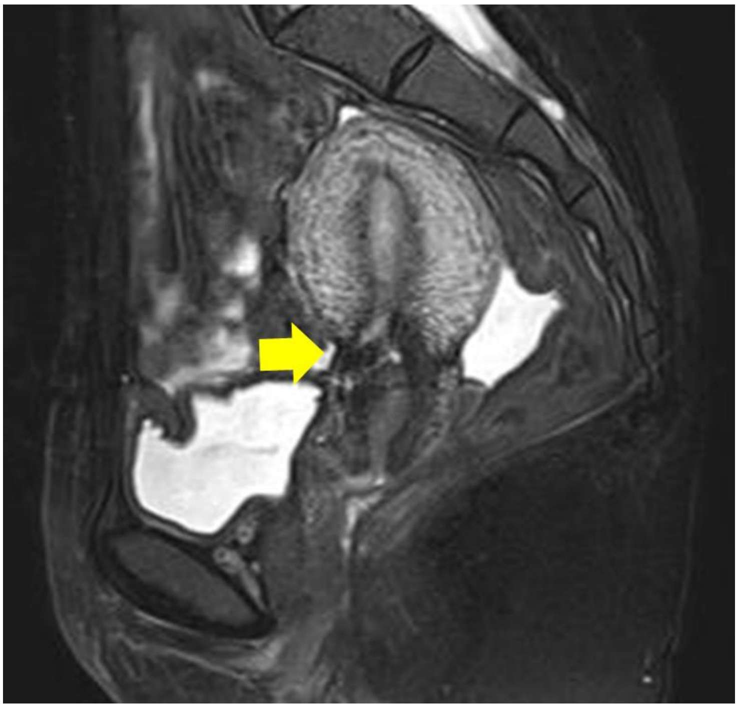

Figure.3　手術時のポート配置

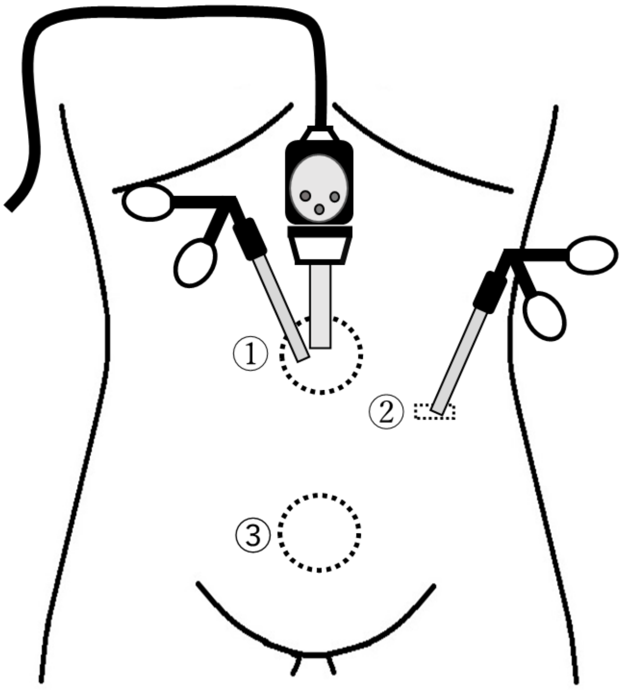

Port placement
①Multichannel port（Umbilical area 25㎜）
②5㎜port(Left side abdomen)
③Multicannel port（Cesarean scar 35㎜）

Figure.4　子宮鏡所見：菲薄部位（矢印）

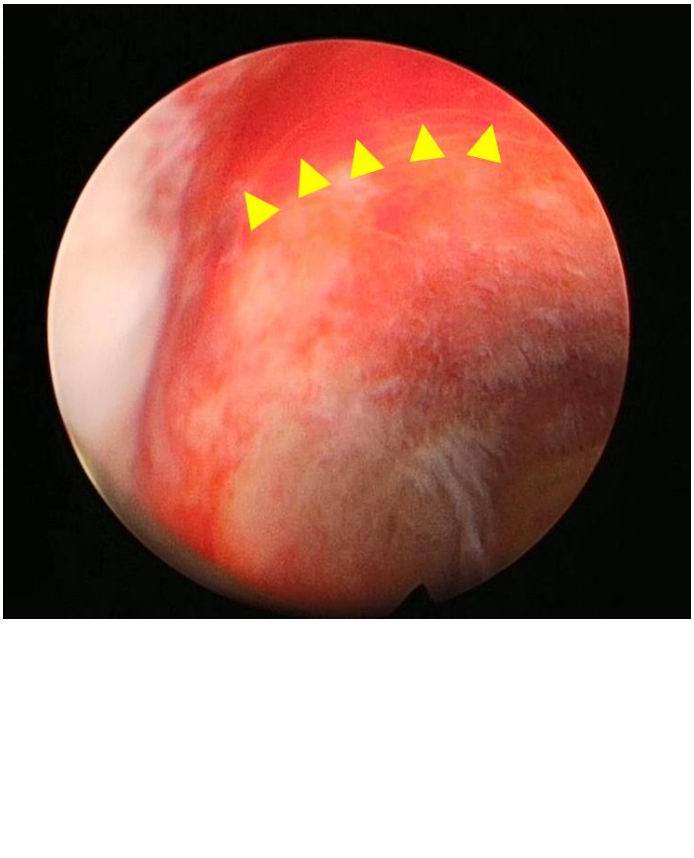

Figure.5　腹腔鏡所見

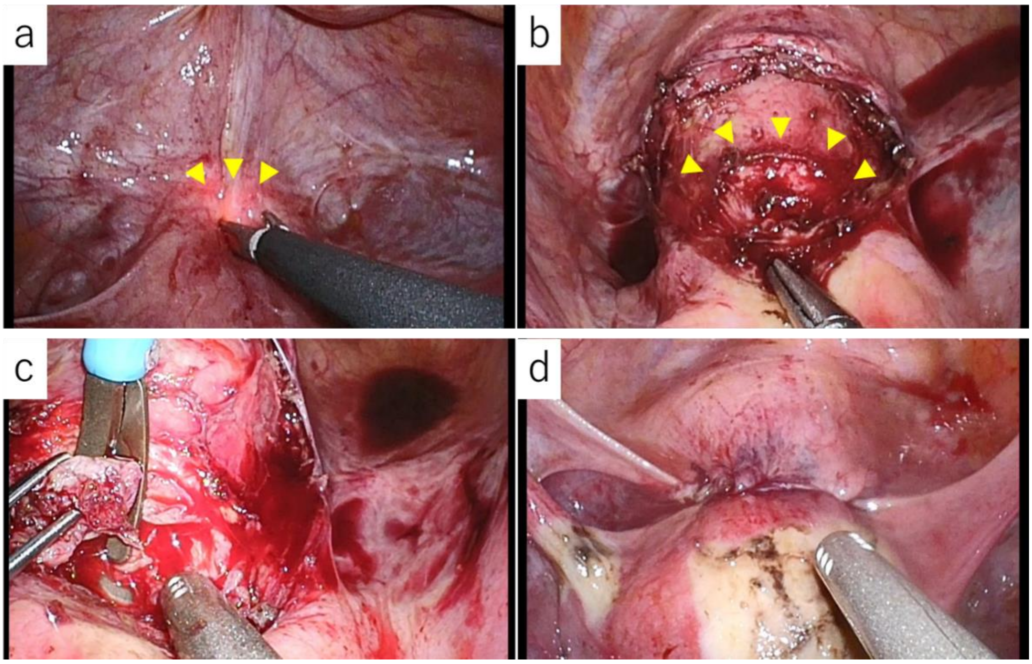

a 子宮鏡の光源を用いた菲薄部位の同定（矢頭）

b 漿膜剥離後の瘢痕部（矢頭）

c 恥骨上からの筋層切開

d 子宮瘢痕部修復術後の所見

Figure.6　単純 MRI T2 強調像矢状断：子宮瘢痕部修復術後 4 か月の residual myometrial thickness (RMT）部（矢印）

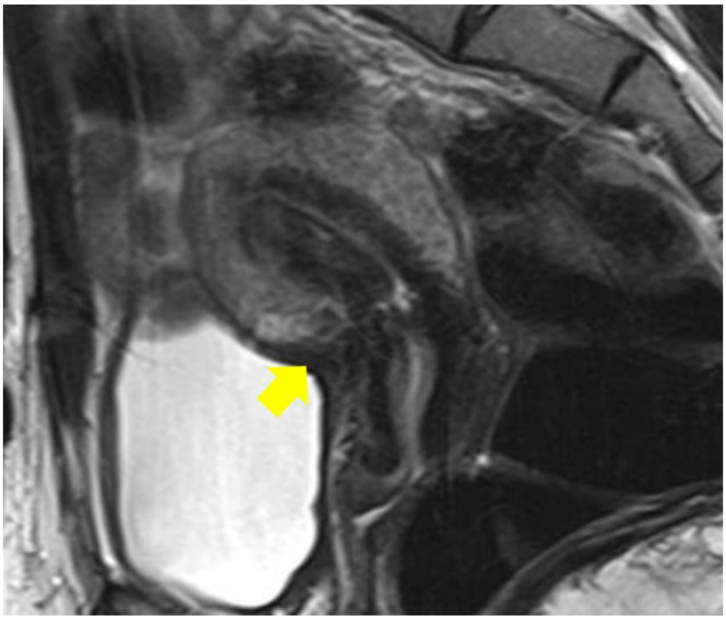

Figure.7　超音波：自然妊娠時の(RMT）部（矢印）

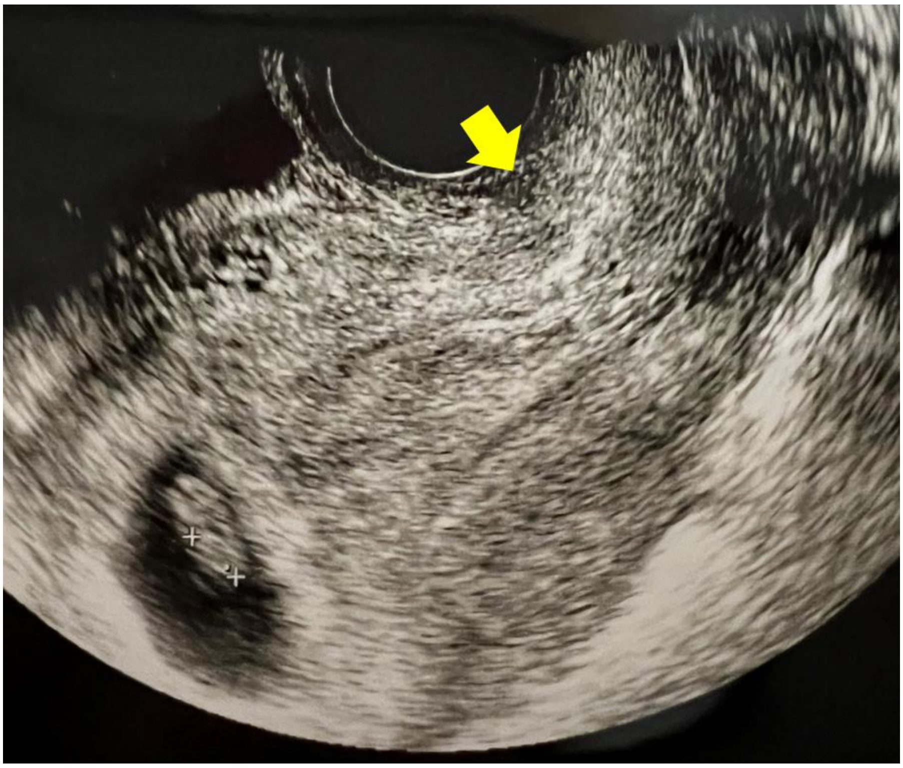

Figure.8　瘢痕部切除操作に対する異なるポート設置によるアプローチ角度の違い

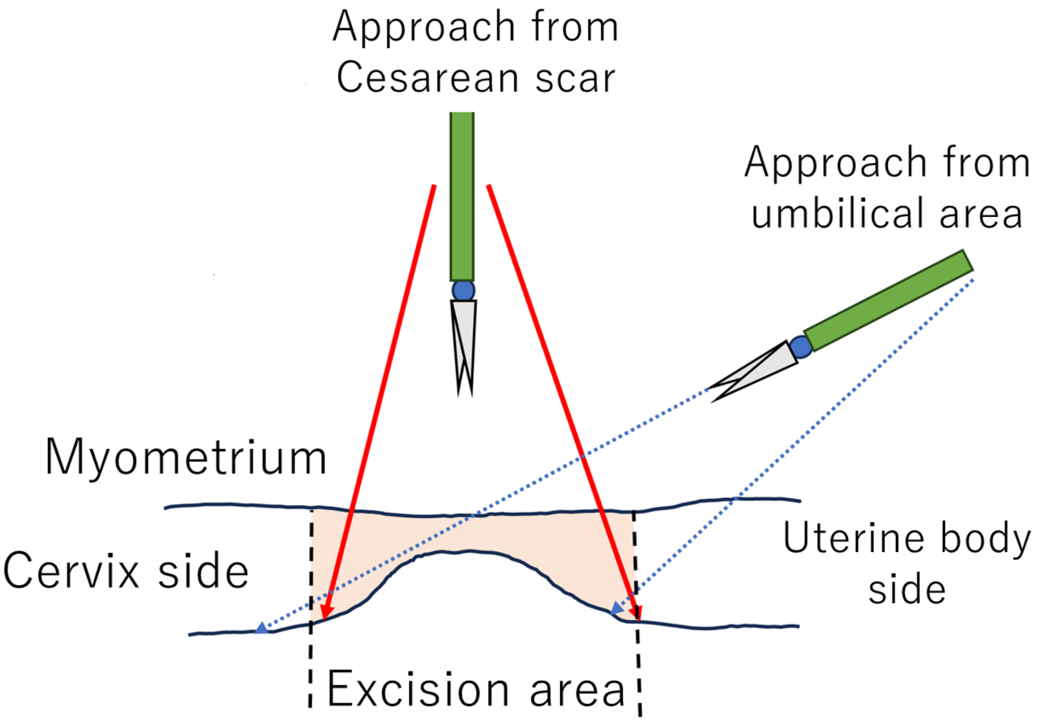

Supplement: Supplementary file 1 — PDF-Japanese [file fmj-11-048-s001.pdf]
